# Optimization of a lumbar interspinous fixation device for the lumbar spine with degenerative disc disease

**DOI:** 10.1371/journal.pone.0265926

**Published:** 2022-04-07

**Authors:** Minhyeok Heo, Jihwan Yun, Hanjong Kim, Sang-Soo Lee, Seonghun Park

**Affiliations:** 1 School of Mechanical Engineering, Pusan National University, Busan, Republic of Korea (South Korea); 2 Institute for Skeletal Aging & Orthopedic Surgery, Hallym University-Chuncheon Sacred Heart Hospital, Chuncheon, Republic of Korea (South Korea); Assiut University Faculty of Medicine, EGYPT

## Abstract

Interspinous spacer devices used in interspinous fixation surgery remove soft tissues in the lumbar spine, such as ligaments and muscles and may cause degenerative diseases in adjacent segments its stiffness is higher than that of the lumbar spine. Therefore, this study aimed to structurally and kinematically optimize a lumbar interspinous fixation device (LIFD) using a full lumbar finite element model that allows for minimally invasive surgery, after which the normal behavior of the lumbar spine is not affected. The proposed healthy and degenerative lumbar spine models reflect the physiological characteristics of the lumbar spine in the human body. The optimum number of spring turns and spring wire diameter in the LIFD were selected as 3 mm and 2 turns, respectively—from a dynamic range of motion (ROM) perspective rather than a structural maximum stress perspective—by applying a 7.5 N∙m extension moment and 500 N follower load to the LIFD-inserted lumbar spine model. As the spring wire diameter in the LIFD increased, the maximum stress generated in the LIFD increased, and the ROM decreased. Further, as the number of spring turns decreased, both the maximum stress and ROM of the LIFD increased. When the optimized LIFD was inserted into a degenerative lumbar spine model with a degenerative disc, the facet joint force of the L3-L4 lumbar segment was reduced by 56%–98% in extension, lateral bending, and axial rotation. These results suggest that the optimized device can strengthen the stability of the lumbar spine that has undergone interspinous fixation surgery and reduce the risk of degenerative diseases at the adjacent lumbar segments.

## Introduction

Lower back pain has various causes, but the degeneration of the intervertebral disc (IVD) is the most common cause of lower back pain [1–3]. When the IVD degenerates, spinal canal stenosis occurs, in which the spinal canal, intervertebral foramina, and nerve root canals are narrowed. If sensory abnormalities in the buttocks or lower extremities, progressive neurologic deficit, and bladder or bowel symptoms appear, spinal fusion is performed along with nerve decompression surgery [4–9]. Spinal fusion is a surgical procedure that uses an implant to connect the vertebral segment of the surgical site to the adjacent segment. This surgical procedure removes a significant portion of the ligaments or muscles that make up the spine [10]. In addition, by fixing the two lumbar segments to one, the movement of the surgical area is restricted. As a result, the movement of adjacent segments increases, and degenerative diseases accelerate in adjacent segments [10–15].

To address the above problems, an interspinous spacer device (ISD) is inserted between the interspinous processes. This increases the height of the lumbar spine segment, which is lowered in patients with spinal stenosis due to the degeneration of the intervertebral disc so that the passage through which the nerve bundle passes is no longer narrowed. The devices developed for this method are X-STOP (Kyphon, Inc., USA), device for intervertebral assisted motion (Medtronic Sofamor Danec, USA), Wallis (Abbott Spine Inc., France), and Coflex (Paradigm Spine LLC, Germany) [16–23]. However, the installation of these devices leads to the removal of soft tissues in the lumbar spine, such as the supraspinous and interspinous ligaments, and an ISD with a higher stiffness than the lumbar spine may cause degenerative diseases in adjacent segments [24,25].

In our previous study, we designed a lumbar interspinous fixation device (LIFD) that addressed the limitations of the ISD and performed a finite element analysis to verify the fundamental performance of the LIFD, in terms of structural and dynamical stabilities. Although the LIFD can be used for the lumbar spine with intervertebral disc diseases, only the L3-L4 lumbar segment was used in this study, and the safety of the interspinous process inserted with the LIFD was not examined by calculating the facet joint force (FJF) in all lumbar segments [26].

Therefore, this study aims to structurally and kinematically optimize an LIFD using a full lumbar finite element model. The optimized LIFD guarantees the structural safety of the spinous processes by FJFs and the kinematically normal behaviors for a range of motions (ROMs) in all lumbar spine segments. This was evaluated after inserting the optimized LIFD into the finite element model of the lumbar spine with the intervertebral disc disease model. Moreover, after installation, the LIFD minimizes degenerative diseases in the adjacent lumbar segments by allowing the lumbar spine to exhibit normal behavior; this can be realized because the interspinous and supraspinous ligaments are not removed during the surgical procedure when the optimized LIFD is employed. The proposed finite element models of the healthy lumbar spine and lumbar spine with degenerative disc disease were also validated via a comparison with the analytical and experimental results of previous studies.

## Materials and methods

### Finite element model of the healthy lumbar spine

**Fig 1:**
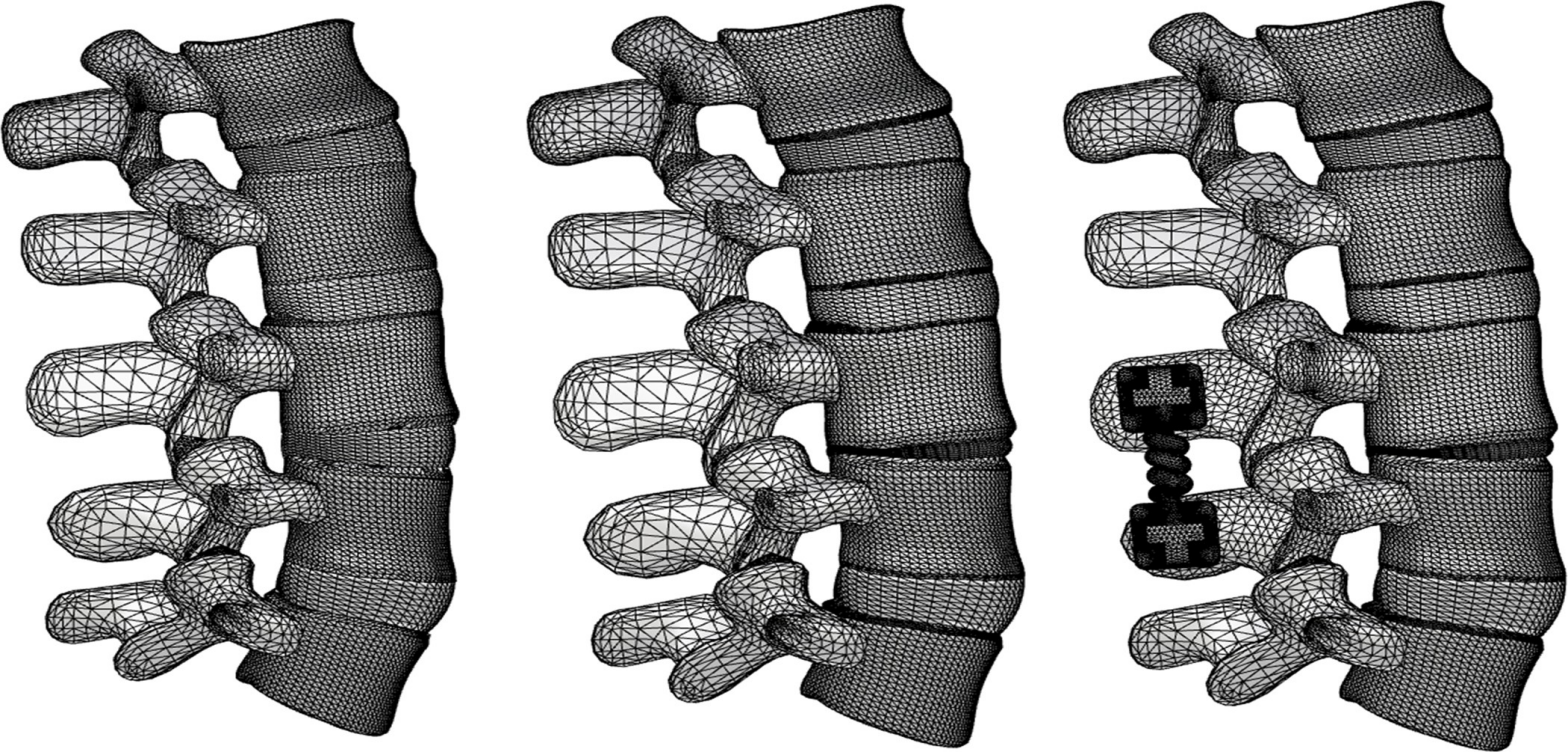
Finite element models of (a) healthy lumbar spine, (b) degenerative lumbar spine with degenerative disc disease, and (c) lumbar spine inserted by the lumbar interspinous fixation device (LIFD).

Lumbar spine data from a human anatomy model (Viewpoint Datalabs, USA) were used to generate the shape of the L1-L5 lumbar spine (Fig 1). The L1-L5 lumbar spine model comprised bone, soft tissues, cartilage, discs, and ligaments. Lumbar vertebrae were created with the cancellous bone, cortical bone, posterior element, and endplate. The cortical bone and endplate were modeled with a thickness of 1 mm [27–32]. The cancellous bone, cortical bone, and endplate were generated with an eight-node hexahedral element (C3D8) and a posterior element with a four-node tetrahedral element (C3D4). The material properties were assumed to be linearly elastic. The facet joint was attached to the upper and lower posterior elements, and the initial upper and lower gaps of the facet joint were modeled as 0.5 mm. For the facet joint, a four-node tetrahedral element (C3D4) with linear elastic properties was employed, and frictionless contact was assumed between the upper and lower facet joints [33,34].

The intervertebral discs between the lumbar spine segments were created with nucleus pulposus, ground substance and annulus fibrosis. The ground substance was modeled as a six-layer, and annulus fibrosis was modeled to surround each layer of ground substance. An eight-node hexahedral element (C3D8H) with a Mooney–Rivlin hyperelastic material property was used as the ground substance. The fluid cavity was formed using a four-node tetrahedral fluid element (SFM3D4) to simulate the fluid behavior of the nucleus pulposus. The annulus fibrosis was modeled as a two-node truss element (T3D2) that resists only tension, and its stiffness increases as the distance from the nucleus pulposus increases [35–37].

The seven types of lumbar ligaments—anterior longitudinal, posterior longitudinal, transverse, interspinous, supraspinous, facet joint capsule, and flavum ligaments—were modeled to be attached to the lumbar spine based on anatomical information. These ligaments were also modeled as a tension-only two-node truss element (T3D2), similar to annulus fibrosis (Table 1) [38,39].

**Table 1:** Material properties of the lumbar spine.

Component	Young’s modulus [MPa]	Poisson ratio	Reference
Cortical bone	12,000	0.3	[29–31]
Cancellous bone	100	0.2	[29–31]
Posterior elements	3,500	0.25	[29–31]
Endplate	23.8	0.4	[29–31]
Cartilage	11	0.4	[40]
Nucleus pulposus	0.0005 mm^2^/N (healty), 0.0995 mm^2^/N (degenerative)		[38]
Ground substance	Hyperelastic Mooney-Rivlin c_1_ = 0.18 c_2_ = 0.045		[35–37]
Annulus fibrosus	Nonlinear, dependant on distance from disc center, 7 layers–criss-cross pattern		[35–37]
Ligament	Nonlinear stress-strain curve		[38,39]

### Validation of the finite element model of the healthy lumbar spine

The healthy lumbar spine finite element model was validated by comparing the ranges of the flexion–extension, lateral bending, axial rotation motion, nucleus pulposus pressure, and FJF available in the literature [34,41–55].

First, the healthy lumbar spine finite element model was verified with a pure moment and pure follower load. The ROM and FJF were calculated by applying a pure moment of 7.5 N∙m to the upper surface of the L1 lumbar vertebra in the directions of flexion, extension, lateral bending, and axial rotation [55]. Next, a follower load of 1000 N was applied to measure the nucleus pulposus pressure of the L4-L5 intervertebral disc [55]. The lower surface of the L5 lumbar vertebra was fixed so that no displacement could be generated in any direction in any of the finite element analyses. Second, a combination of moment and follower load was applied, and the ROM, nucleus pulposus pressure, and FJF were measured. These loading conditions were referenced from previous studies that measured in vivo experiments (Table 2) [56–58].

**Table 2:** Loading and moment conditions of four lumbar spine motions.

	Follower load (N)	Moment (Nm)	References
Flexion	1175	7.5	[56]
Extension	500	7.5	[56]
Lateral bending	700	7.8	[57]
Axial rotation	720	5.5	[58]

### Finite element model of the lumbar spine with degenerative disc disease

The lumbar finite element model with the degenerative disc was developed using the healthy lumbar spine finite element model with reference to previous studies, in which degenerative disc disease caused changes in the disc height. A degenerative disc was assumed to be present between the L3-L4 lumbar vertebrae. In addition, the changes in the material properties and shape of the degenerative disc were referenced in the literature [1,38].

In this study, compared with a healthy disc, the degenerative disc for the LIFD insertion was assumed to have a 60% reduction in height (Fig 1). The material properties of the nucleus pulposus were applied by referring to the results of previous studies showing that when the height of the disc was reduced by 60%, the compressibility of the healthy nucleus pulposus increased from 0.0005 mm^2^/N to 0.0995 mm^2^/N [38,48]. This reduction in disc height caused the anterior, posterior, transverse, flavum, capsular ligaments, and annulus fibrosis to buckle; however, the interspinous and supraspinal ligaments were prestressed without buckling. Therefore, by offsetting the nonlinear stress–strain curve, prestress was applied to the interspinous and supraspinal ligaments, whereas the buckled ligaments and annulus fibrosis were made to work according to the original nonlinear stress–strain curve when they achieved their original lengths [38].

### Validation of the finite element model of the lumbar spine with the degenerative disc disease

The validation of the lumbar finite element model with the degenerative disc was based on the results of previous studies, which verified the finite element model only in the L3-L4 lumbar region. Therefore, we used only the L3-L4 lumbar region for comparison with the results of the present study. The lower surface of the L4 lumbar vertebra was fixed, and a pure moment of 10 N∙m was applied to the upper surface of the L3 lumbar vertebra in the directions of flexion, extension, lateral bending, and axial rotation. The finite element model of this study was verified by comparing the ROM with previous findings [59].

### Optimum design and validation of the LIFD

**Fig 2:**
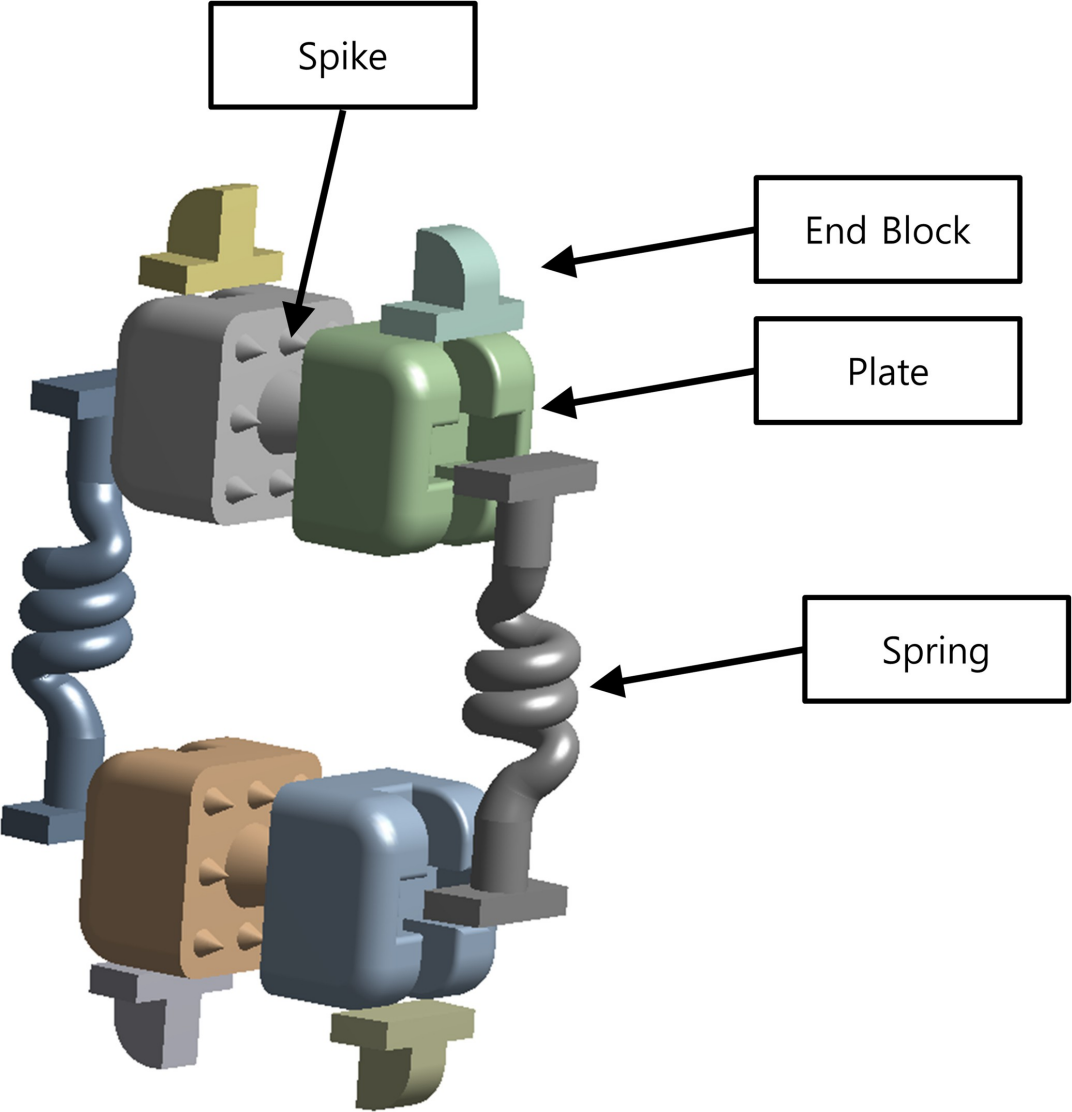
Components of the lumbar interspinous fixation device (LIFD).

The LIFD spring component designed in our previous study was optimized using the Taguchi method (Fig 2). The number of turns of the active coils of the spring and the wire diameter of the spring were selected as design variables, and the spring stiffness was fixed at 20 kN based on our previous results [26]. The number of turns of the active coils of the spring and the wire diameter of the spring were calculated as follows:

$$k=\frac{P}{\delta }=\frac{Gd^{4}}{8ND^{3}}$$ (1)

where G denotes the shear modulus, d is the wire diameter of the spring, D is the mean diameter of the spring, and N is the number of turns of the active coils of the spring.

An L9 (23) orthogonal array developed by Taguchi was adopted for the optimum design of the LIFD. The L9 array has two variables, each with three levels; hence, nine finite element analyses were conducted to optimize the design variables (Table 3). Next, the LIFD was inserted into the L1-L5 model with the degenerative disc in the L3-L4 lumbar vertebrae to create the optimal design. To simulate the extension of the LIFD itself and the LIFD-inserted spinous process, the lower surface of the L5 lumbar vertebra was fixed such that no displacement could occur in any direction. A moment of 7.5 N∙m in the extension direction and a follower load of 500 N were applied to the upper surface of the L1 lumbar vertebra.

**Table 3:** Set of factors for Taguchi Array L9.

Experiment number	Wire diameter (mm)	Number of turns
1	3	2
2	3	2.5
3	3	3
4	3.5	2
5	3.5	2.5
6	3.5	3
7	4	2
8	4	2.5
9	4	3

As described above, after selecting the optimal values of the design variables of the LIFD in the extension motion, this optimally designed LIFD was inserted into the lumbar spine model with the degenerative intervertebral disc. Next, the loading conditions listed in Table 2 were applied. The performance of the optimally designed LIFD was examined in terms of the ROM and FJF of both the healthy lumbar spine and lumbar spine, with the degenerative intervertebral disc in each behavior.

## Results

### Validation of the finite element model of the healthy lumbar spine

**Fig 3:**
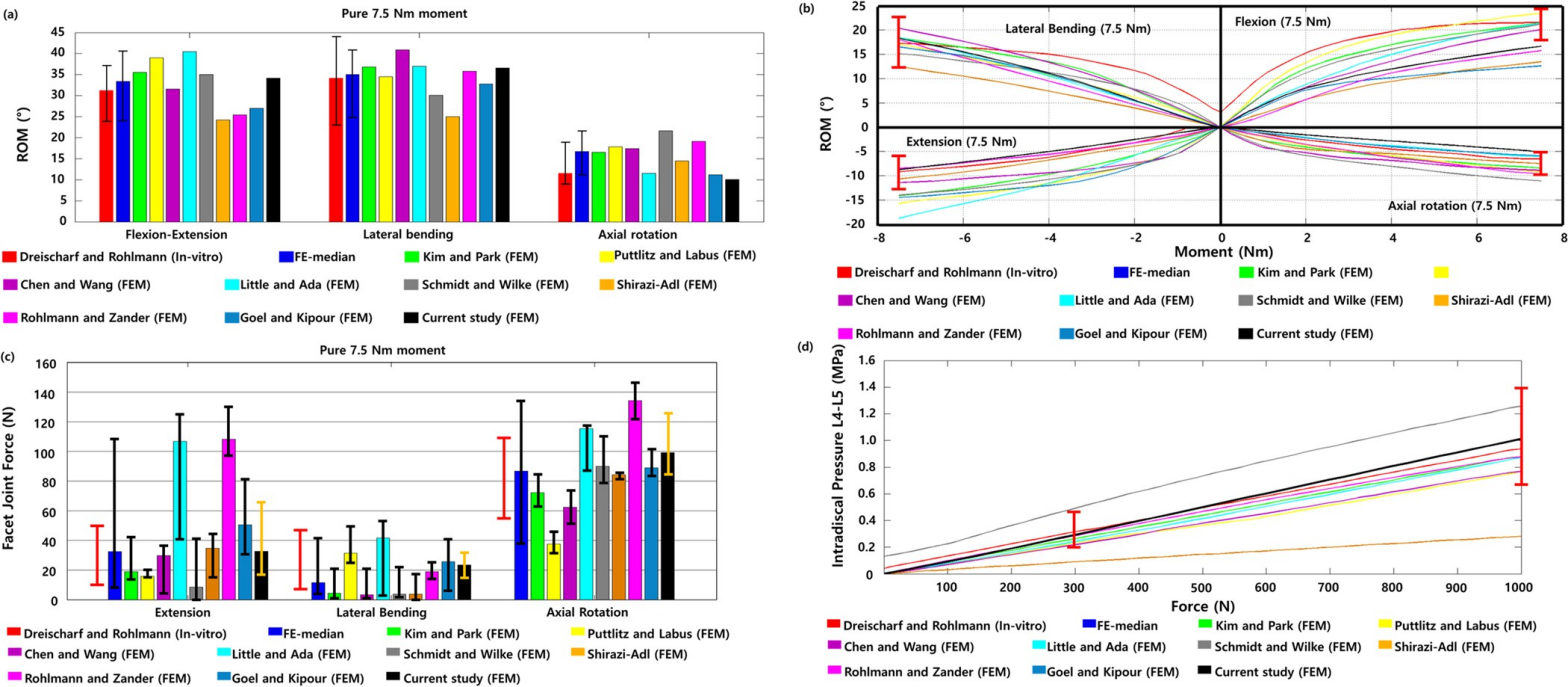
(a) Range of motion (ROM) of the healthy lumbar spine from entire L1 to L5 segments (i.e., healthy L1-L5 lumbar spine) under pure 7.5 N∙m moment (The red bar is the median value of previous in vitro experimental findings, and other bars represent previous and current findings of finite element analyses). (b) ROM of the healthy L1-L5 under pure moment of 0 to 7.5 N∙m (The red range bar represents previous findings of in vitro experiments under 7.5 N∙m moment). (c) Facet joint forces (FJFs) of the healthy L1-L5 under pure 7.5 N∙m moment (The red bar is the median value of previous in vitro experimental findings, and other bars represent previous and current findings of finite element analyses). (d) Nucleus pulposus pressure of the L4-L5 intervertebral disc under follower load of 0 to 1000 N (The red range bar represents previous findings of in vitro experiments under 300 N and 1000 N follower loads).

The ROM of the healthy lumbar spine for the entire L1 to L5 segments (i.e., L1-L5) during flexion, extension, lateral bending, and axial rotation under a pure moment was compared with the results of previous studies. The ROM was 20.2° in flexion, 14° in extension, 39.2° in lateral bending, and 10.6° in axial rotation [Fig 3(A) and 3(B)]. Next, the FJF of the L1-L5 was measured as 32.6 N in extension, 23.5 N in lateral bending, and 99.3 N in axial rotation [Fig 3(C)]. The current results of ROM and FJF were compatible with previous findings of finite element analysis and in vitro experiments.

The nucleus pulposus pressure of the intervertebral disc between the L4-L5 under a pure follower load was 1.01 MPa, consistent with previous findings of finite element analysis and in vitro experiments [Fig 3(D)].

**Fig 4:**
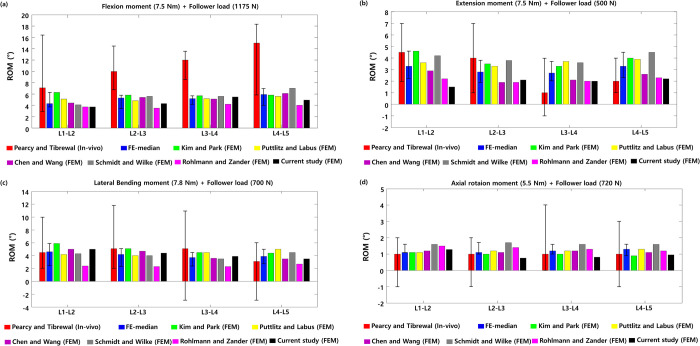
Range of motions (ROM) between two healthy lumbar spine segments was measured under a combination of (a) flexion moment (7.5 N∙m) and follower load (1175 N), (b) extension moment (7.5 N∙m) and follower load (500 N), (c) lateral bending moment (7.8 N∙m) and follower load (700 N), and (d) axial rotation moment (5.5 N∙m) and follower load (720 N) (The red bar is the median value of previous in vivo experimental findings, and other bars represent previous and current findings obtained from finite element analyses).

Next, the ROM between the two healthy lumbar spine segments was measured under moment and follower loads. The ROMs between the L1-L2, L2-L3, L3-L4, and L4-L5 lumbar segments were 3.73°, 4.29°, 5.46°, and 4.91°, respectively, during flexion under a 7.5 N∙m moment and 1175 N follower load. The ROMs were 3.66°, 2.27°, 2.86°, and 3.55°, respectively, during extension under a 7.5 N∙m moment and 500 N follower load. The ROMs were 5.53°, 2.86°, 6.04°, and 5.2°, respectively, during lateral bending under a 7.8 N∙m moment and 700 N follower load. The ROM was 1.28° for L1-L2, 0.76° for L2-L3, 1.26° for L3-L4, and 0.95° for L4-L5 during axial rotation under a 5.5 N∙m moment and 720 N follower load, as shown in Fig 4.

**Fig 5:**
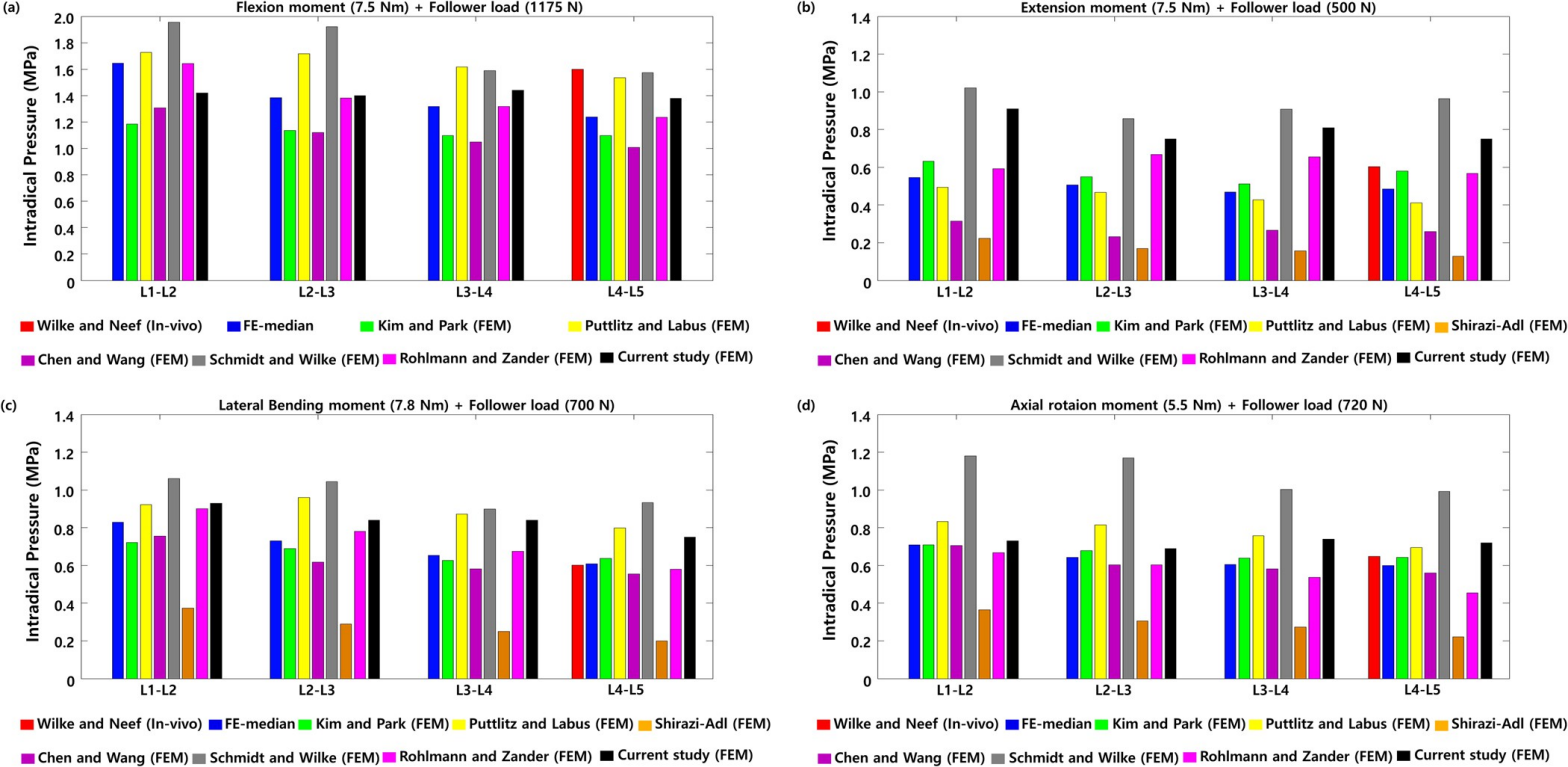
Nucleus pulposus pressure of the L4-L5 intervertebral disc measured under a (a) flexion moment (7.5 N∙m) and follower load (1175 N), (b) extension moment (7.5 N∙m) and follower load (500 N), (c) lateral bending moment (7.8 N∙m) and follower load (700 N), and (d) axial rotation moment (5.5 N∙m) and follower load (720 N) (The red bar is the median value of previous in vivo experimental findings, and other bars represent previous and current findings obtained from finite element analyses).

**Fig 6:**
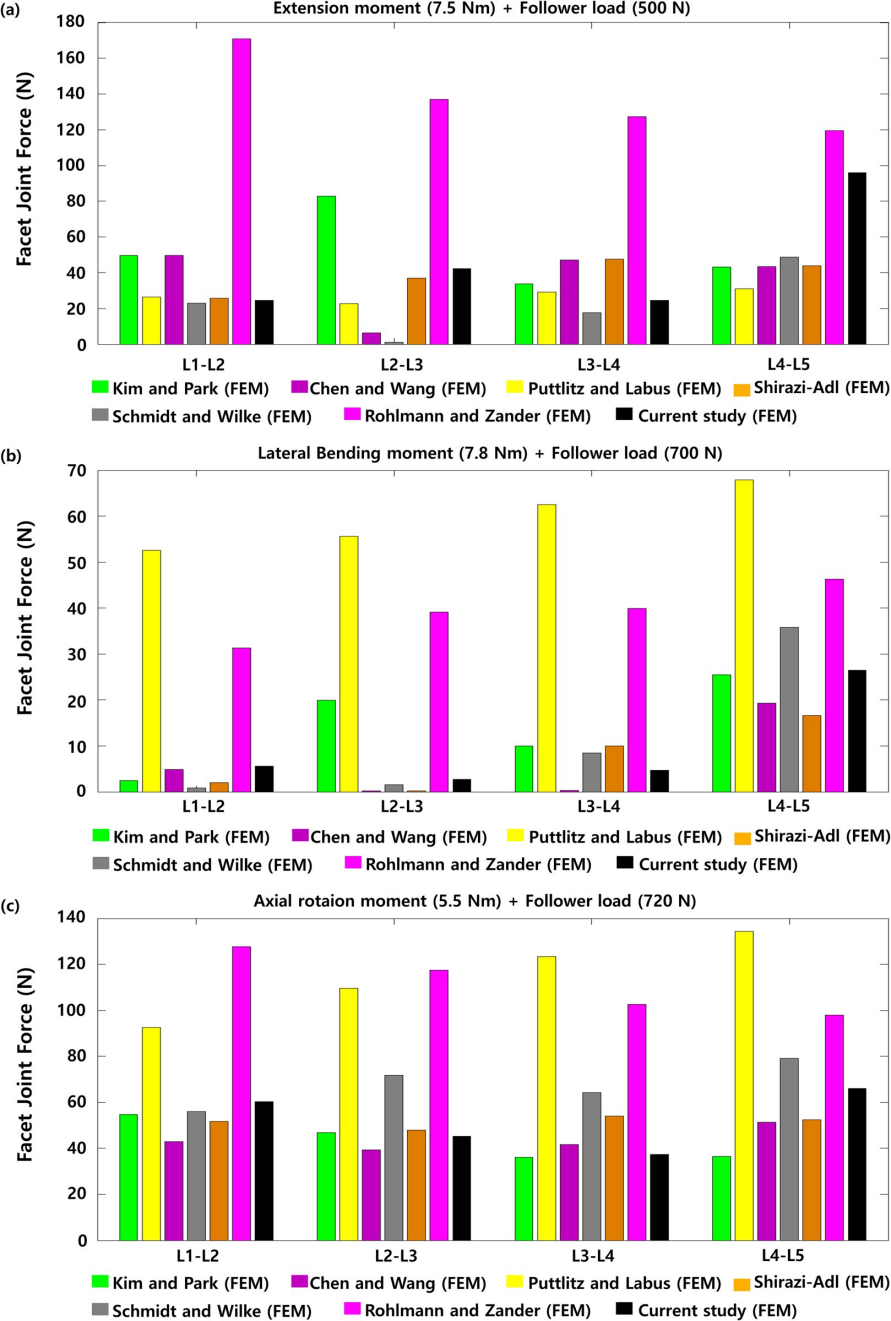
Facet joint force (FJF) of the two healthy lumbar spine joints under a combination of (a) flexion moment (7.5 N∙m) and follower load (1175 N), (b) extension moment (7.5 N∙m) and follower load (500 N), (c) lateral bending moment (7.8 N∙m) and follower load (700 N), and (d) axial rotation moment (5.5 N∙m) and follower load (720 N).

The NP pressures of the intervertebral discs between the healthy L1-L2, L2-L3, L3-L4, and L4-L5 lumbar segments during flexion were 1.42 MPa, 1.4 MPa, 1.44 MPa, and 1.38 MPa, respectively. Those pressures during extension were 0.91 MPa, 0.75 MPa, 0.81 MPa, and 0.75 MPa, respectively. The nucleus pulposus pressures were 0.93 MPa, 0.84 MPa, 0.84 MPa, and 0.75 MPa, respectively, during lateral bending. The nucleus pulposus pressures were 0.73 MPa, 0.69 MPa, 0.74 MPa, and 0.72 MPa during axial rotation (Fig 5). The FJFs between the healthy L1-L2, L2-L3, L3-L4, and L4-L5 lumbar segments were 24.6 N, 42.4 N, 24.6 N, and 96.1 N, respectively, during extension; 5.64 N, 2.76 N, 4.75 N, and 26.5 N, respectively, during lateral bending; 60.3 N, 45.2 N, 37.3 N, and 66.0 N, respectively, during axial rotation (Fig 6). The ROMs and nucleus pulposus pressures between the two lumbar spine segments were consistent with previous findings of finite element analysis and in vivo experiments.

### Validation of the finite element model of the lumbar spine with the degenerative disc disease

**Fig 7:**
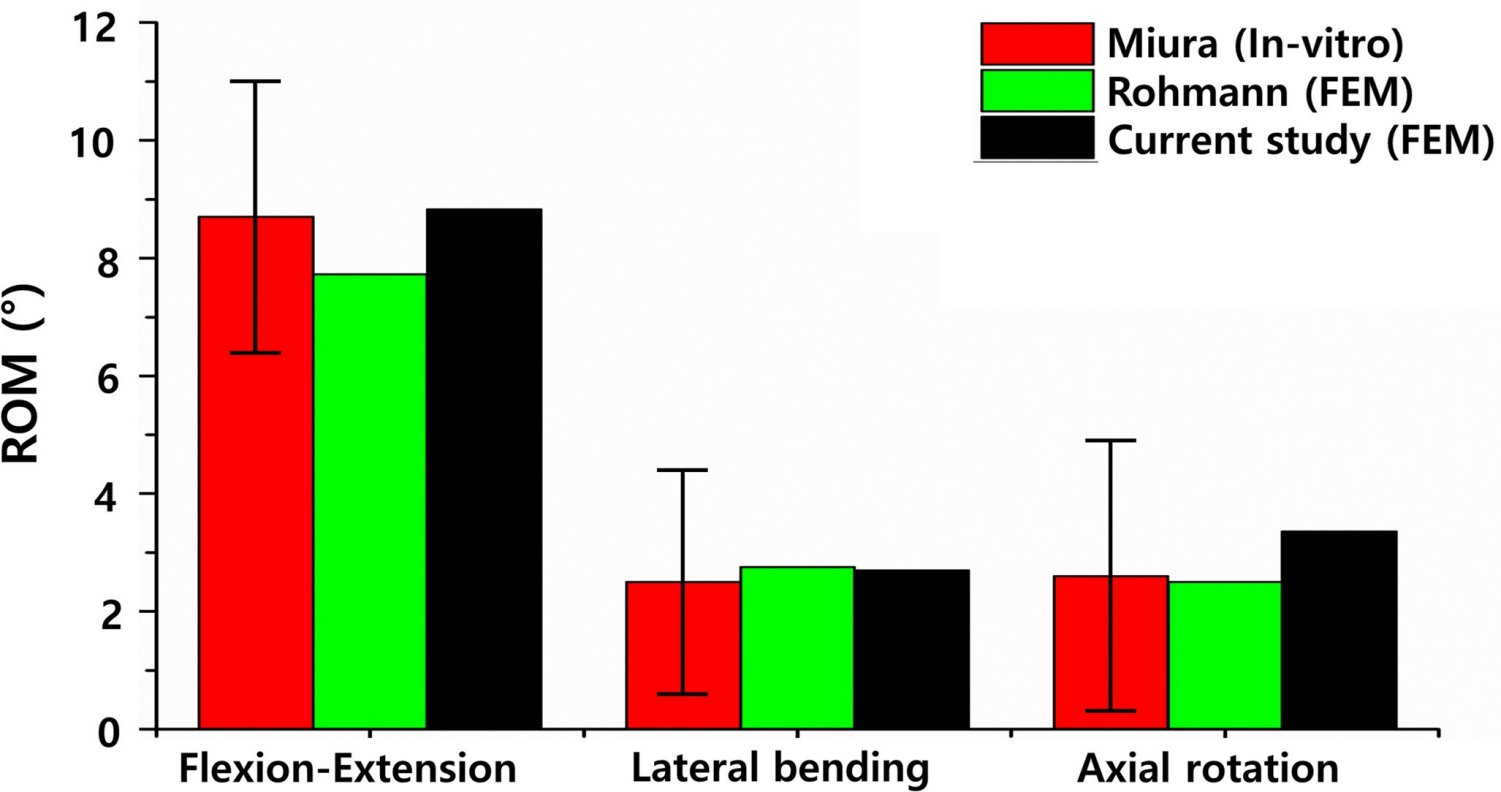
Range of motion (ROM) of the degenerative L3-L4 lumbar spine with degenerative disc disease under pure moment (10 N∙m) in flexion–extension, lateral bending, and axial rotation.

The ROMs of the healthy L3-L4 lumbar spine segment model with a healthy disc model and degenerative L3-L4 lumbar spine segment model with degenerative disc model during flexion, extension, lateral bending, and axial rotation under a pure moment of 10 N∙m were compared with the previous findings of experimental studies and finite element analyses. The ROMs between the healthy L3-L4 was 6.2°, 2.5°, 6.65°, and 2.74° during flexion, extension, lateral bending, and axial rotation, respectively. The ROMs between the degenerative L3-L4 was 5.38°, 3.45°, 2.7°, and 3.36° during flexion, extension, lateral bending, and axial rotation, respectively (Fig 7). These ROMs were consistent with previous in vitro experimental results in flexion, extension, lateral bending, and axial rotation at a moment of 10 N∙m.

### Optimum design and validation of the LIFD

The maximum stresses of the LIFD were calculated according to changes in the number of turns of the LIFD spring and the wire diameter during extension under a 7.5 N∙m moment and 500 N follower load. The ROMs and FJFs were calculated between the healthy L3-L4 lumbar spine model and the degenerative L3-L4 model as well as the degenerative L3-L4 lumbar spine with the LIFD inserted.

**Fig 8:**
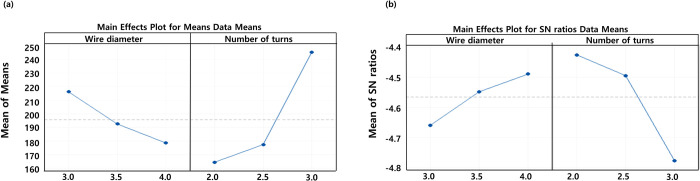
(a) Main effects plot of the means and (b) main effects plot of the S/N ratios (larger is better) for the maximum stress in the LIFD.

**Fig 9:**
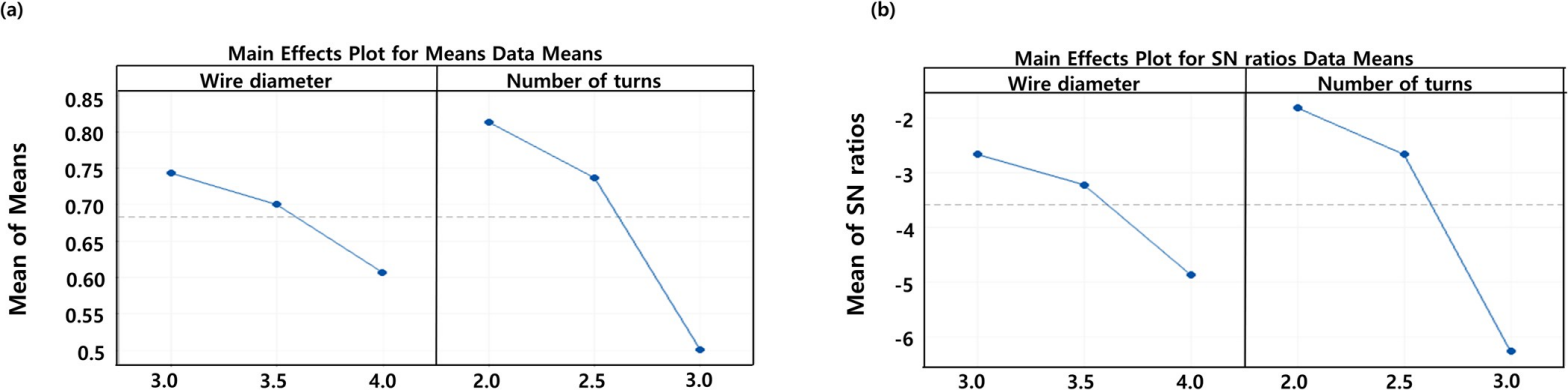
(a) Main effects plot of the means and (b) main effects plot of the S/N ratios (larger is better) for the Range of motion (ROMO of the L3-L4 lumbar spine segment.

When the spring wire diameter was 3 mm, the maximum stresses of the LIFD in extension were 191 MPa, 195.7 MPa, and 261.5 MPa with the number of spring turns at 2, 2.5, and 3, respectively. Using the same spring design variable values as above, the ROMs between the LIFD-inserted L3-L4 lumbar spine model were 0.85°, 0.77°, and 0.61°, respectively. When the spring wire diameter was 3.5 mm, the maximum stresses of the LIFD in extension were 153.2 MPa, 172.5 MPa, and 251.6 MPa, for the number of spring turns of 2, 2.5, and 3, respectively. In addition, for the same spring design variable values as above, the ROMs were 0.82°, 0.73°, and 0.55°, respectively. Finally, when the spring wire diameter was 4 mm, the maximum stresses of the LIFD in extension were 148.9 MPa, 163.7 MPa, and 223 MPa, for the number of spring turns of 2, 2.5, and 3, respectively. For the same spring design parameter values as above, the ROMs were 0.77°, 0.71°, and 0.34°, respectively. The maximum stresses of the LIFD and ROMs of the LIFD-inserted L3-L4 lumbar spine decreased with increasing spring wire diameter, and the number of spring turns of the LIFD (Figs 8 and 9).

**Fig 10:**
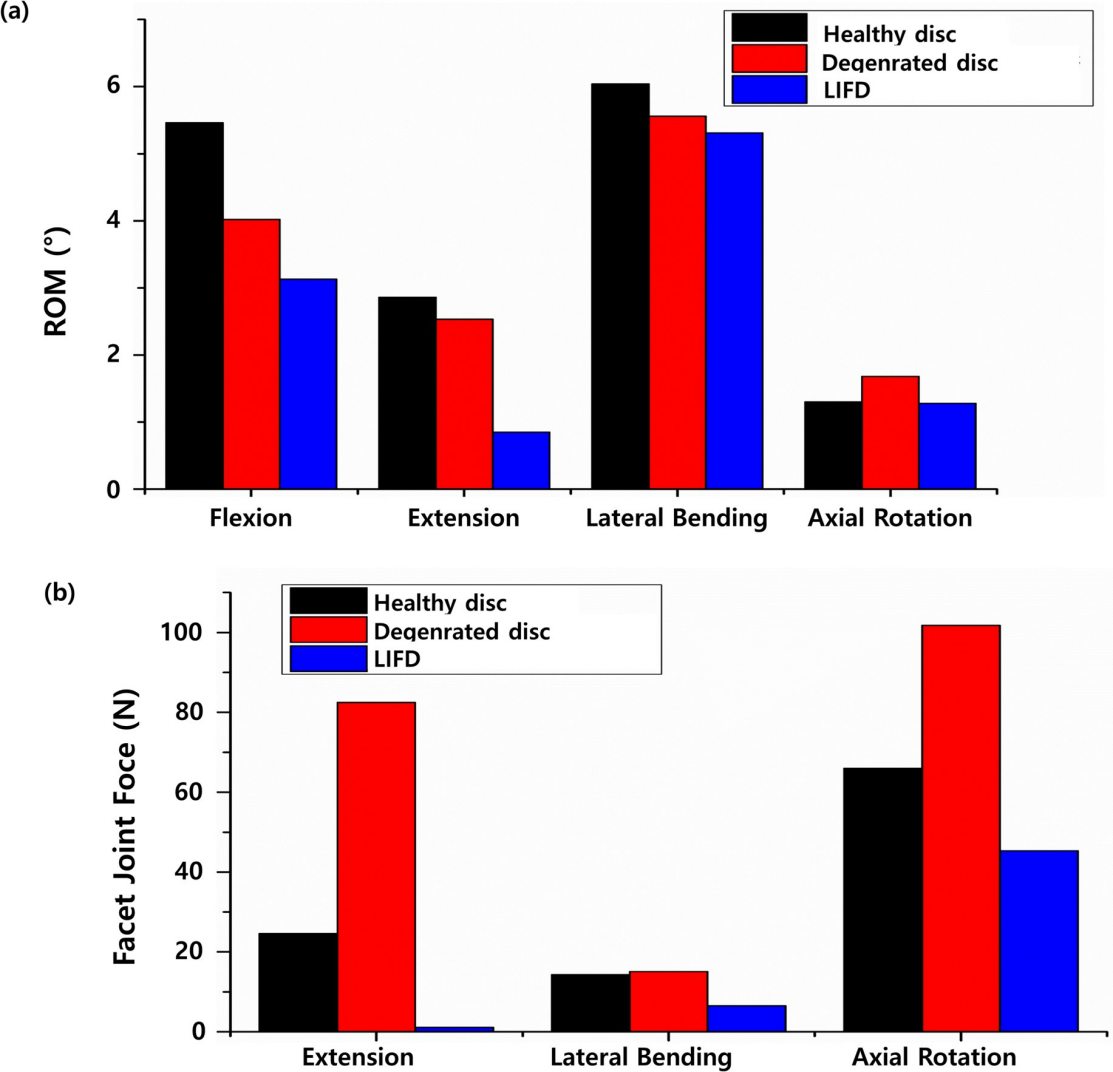
Finite element analysis results of the healthy L3-L4 lumbar spine, the degenerative L3-L4 with degenerative disc disease, the L3-L4 lumbar spine inserted by the lumbar interspinous fixation device (LIFD) in flexion (1175-N follower load and 7.5- N∙m moment), extension (500 N follower load and 7.5 N∙m moment), lateral bending (700 N follower load and 7.8 N∙m moment), and axial rotation motion (720 N follower load and 5.5 N∙m moment): (a) range of motion (ROM) (b) facet joint force (FJF).

To analyze the performance of the optimally designed LIFD, both the ROMs and FJFs of the healthy L3-L4, degenerative L3-L4, and LIFD-inserted L3-L4 were computed in flexion, extension, lateral bending, and axial rotation motion under the loading conditions listed in Table 2. The ROMs of the healthy L3-L4 in flexion, extension, lateral bending, and axial rotation motion was 5.46°, 2.86°, 6.04°, and 1.3°, respectively. The ROMs of the degenerative L3-L4 was 4.02°, 2.53°, 4.05°, and 1.68°, respectively. The FJFs of healthy L3-L4 were 24.6 N, 14.3 N, and 66 N in extension, lateral bending, and axial rotation motion, respectively. The FJFs of the degenerative L3-L4 were 82.5 N, 15.1 N, and 101.8 N, respectively. The ROMs of the LIFD-inserted L3-L4 was 2.65°, 0.85°, 4.92°, and 1.28° in flexion, extension, lateral bending, and axial rotation motion, respectively. The FJFs of the LIFD-inserted L3-L4 were 1.14 N, 6.5 N, and 45.3 N in extension, lateral bending, and axial rotation motion (Fig 10).

Based on the above results, the optimal design variable values were selected by considering the maximum stress and ROM values. As the maximum stress was minimized, the values of the spring wire diameter and number of spring turns were 4 mm and 2, respectively. However, as the ROM was maximized, the values of the spring wire diameter and number of spring turns were 3 mm and 2, respectively. When the ROM is restricted, compensatory movements may occur in the lumbar segment above or below the operated lumbar segment, leading to degenerative disease of the lumbar disc above or below the operated lumbar segment. Therefore, we chose a spring wire with a diameter of 3 mm and two turns, focusing on the ROM of the lumbar spine rather than the maximum stress of the LIFD. Using these design variables, the maximum stress computed in the LIFD under 7.5 N∙m moment and 500 N follower loads was 191 MPa. This maximum stress value corresponded to 20.1% of the LIFD material yield strength (i.e., 950 MPa) and resulted in a safety factor of 5 [60]. Therefore, the spring wire with a diameter of 3 mm and two turns in the LIFD satisfy both the mechanical stability and maximum ROM value.

## Discussion

In the current study, a finite element model of a healthy lumbar spine was developed based on the human anatomy model and verified via a comparison with existing in vitro and in vivo experimental data and finite element analysis results. A finite element model of a degenerative lumbar spine was also created with a degenerative intervertebral disc model degenerated at various levels and verified via a comparison with the results of previous studies. After inserting the LIFD into the L3-L4 lumbar spine segments with the degenerative disc, the maximum stress at the interspinous process and the LIFD, ROM, FJF, and nucleus pulposus pressure were computed in the extension motion. These models were used to design the LIFD optimally. Finally, the optimally designed LIFD was inserted into the degenerative lumbar spine model with a degenerative disc, and then the ROM and FJFs between the LIFD-inserted L3-L4 in flexion, extension, lateral bending, and axial rotation were measured to validate the performance of the LIFD.

### Validation of the finite element model of the lumbar spine

The ROMs of the healthy lumbar spine model in flexion, extension, lateral bending, and axial rotation motion was consistent with previous in vitro experimental findings [Fig 3(A) and 3(B)]. The FJFs for the extension and axial rotation motions were within the range of the previous in vitro experimental findings [Fig 3(C)]. The FJF values varied between different finite element models constructed by different researchers depending on the shape modeling of the facet joint cartilage and the applied friction conditions. The pressure of the nucleus pulposus of the intervertebral disc was also within the range of previous findings obtained with other lumbar finite element models but was approximately 14% to 25% smaller or greater than the experimental values in vivo. However, considering that the in vivo test results were measured in a single subject under maximal voluntary motion, a difference of approximately 25% between the experimental results and the analytical results can be evaluated as a reasonable result [54]. In this study, both the FJF and nucleus pulposus pressure values of the L4-L5 intervertebral disc were also consistent with the range of in vitro experimental results [Fig 3(D)].

For both the healthy and degenerative L3-L4 lumbar spine models, the ROMs calculated in the directions of flexion–extension, lateral bending, and axial rotation under a 10 N∙m moment were also consistent with previous in vitro experimental findings. In this study, the interspinous and supraspinous ligaments were prestressed owing to the loss of intervertebral disc height when modeled as degenerative discs. Therefore, the stiffness of the degenerative lumbar spine to resist flexion–extension and lateral bending motion increased, and hence, the ROM of the degenerative L3-L4 lumbar spine decreased. Moreover, consistent with the literature findings, all ligaments except the interspinous and supraspinous ligaments were buckled in the degenerative L3-L4, resulting in an increase in the ROM of axial rotation compared to the healthy L3-L4 [38,59]. These results suggest that the finite element model of the lumbar spine constructed in this study is suitable for use in biomechanical analysis because it reflects the physiological characteristics of the human lumbar spine.

### Optimum design and validation of the LIFD

The maximum stress calculated in the spinous process in the extension motion increased with an increasing spring wire diameter and an increasing number of spring turns in the LIFD, whereas the ROM of the L3-L4 lumbar spine decreased. As the spring wire diameter and number of spring turns increased, the structural stiffness of the spring increased in the extension motion, reducing the deformation of the LIFD. In the extension motion, the ROM of–L3-L4 tended to increase when the gap between the interspinous processes decreased, while the ROM tended to decrease when the LIFD deformation decreased. The maximum stress in the LIFD increased with a decreasing spring wire diameter and an increasing number of spring turns. This is because an increase in the spring wire diameter results in the distribution of the LIFD load, and a decrease in the number of spring turns results in a decrease in the spring stiffness, and hence, an increase in the spring deformation. This result also indicates that a decrease in the number of spring turns decreases the load that prevents the L3-L4 lumbar spine movement in the extension and decreases the maximum stress produced in the LIFD.

The LIFD was inserted into the degenerative L3-L4 lumbar segment to verify the performance of the optimally designed LIFD. Comparing the ROM and FJF before and after insertion, the ROM decreased by 22% in flexion and 66% in extension, while the FJF decreased by 98.6% in extension under the same conditions. The significant decrease in FJF and ROM in the flexion and extension motions indicates that the spring structure of the LIFD has a significant influence on tension and compression. Moreover, the result of a 1.54% decrease in ROM after LIFD insertion in the axial rotation motion compared with healthy L3-L4 lumbar segments also indicates that unstable behavior can be recovered in the axial rotation motion. The maximum stress (~24.5 MPa) of the spinous process for the LIFD-inserted L3-L4 lumbar spine under a 7.5 N∙m moment and 500 N follower load, which is significantly lower than the bone fracture stress (~213 MPa) reported in the literature [61–63], demonstrates the mechanical integrity of the current LIFD design. Although a decrease in ROM is expected after LIFD insertion surgery, this has been observed in previous LIFD studies in the literature reporting pain reduction in the intervertebral disc and posterior joints [20,21]. Therefore, the insertion of the currently designed LIFD in a patient with an intervertebral disease can significantly reduce FJF and intervertebral pain.

In previous studies, the performance of the interspinous devices was evaluated using a finite element analysis by inserting the devices into a healthy lumbar finite element model. However, when a healthy lumbar finite element model is used, the physical properties and deformation information of degenerative discs are not used. For this reason, changes in the ROM and force of the facet joint due to degenerative intervertebral discs cannot be reflected. Therefore, the effectiveness of the interspinous device cannot be confirmed in degenerative intervertebral discs. In this study, a design of LIFD was proposed by selecting the optimal values of the spring wire diameter as well as the number of spring turns for the spring in the LIFD. The performance of the LIFD was confirmed using the lumbar finite element model with the degenerative disc model implemented in this study. However, the limitations of the current study are as follows. The information on degenerative disc using a finite element analysis was reflected, but lesions such as osteopenia, osteoporosis, and lumbar spine spondylolisthesis were not reflected in patients requiring surgery. Bone fracture stress in patients with osteopenia or osteoporosis is lower than the reported bone fracture stress; therefore, studies that reflect this are required. In addition, the performance of the current LIFD was evaluated only through finite element analysis and not through rig tests using material testing machines.

## Conclusions

In this paper, we proposed a design for LIFD by selecting the optimal values of the spring wire diameter; and the number of spring turns for the spring in the LIFD from a dynamic perspective. The result that the FJF of the LIFD-inserted lumbar spine with the degenerative disc decreased by approximately 55% to 98% in extension, lateral bending, and axial rotation suggests that the optimally designed LIFD in this study can reduce pain caused by posterior joint lesions. In future studies, fatigue tests and analyses should be performed with the LIFD to validate the durability of the LIFD in the human body and investigate changes in performance and safety after long-term use.

## Supporting information

S1 Dataset (XLSX)
